# Aggregated occurrence records of the invasive alien striped field mouse (*Apodemusagrarius* Pall.) in the former USSR

**DOI:** 10.3897/BDJ.9.e69159

**Published:** 2021-06-22

**Authors:** Lyudmila A Khlyap, Vladimir Dinets, Andrey A Warshavsky, Fedor A Osipov, Natalia N Dergunova, Varos G Petrosyan

**Affiliations:** 1 A.N. Severtsov Institute of Ecology and Evolution of the Russian Academy of Sciences, Moscow, Russia A.N. Severtsov Institute of Ecology and Evolution of the Russian Academy of Sciences Moscow Russia; 2 University of Tennessee, Knoxville, United States of America University of Tennessee Knoxville United States of America; 3 Kean University, Union, United States of America Kean University Union United States of America

**Keywords:** invasive species, distribution, occurrence records, agrophil, hemisinanthrope, zoonotic diseases

## Abstract

**Background:**

Open access to occurrence records of the most dangerous invasive species in a standardised format have important potential applications for ecological research and management, including the assessment of invasion risks, formulation of preventative and management plans in the context of global climate and land use changes in the short and long perspective. The striped field mouse (*Apodemusagrarius* Pallas, 1771) is a common species in the temperate latitudes of the Palaearctic. Due to land use and global climate changes, several waves of expansion of the range of this species have been observed or inferred. By intrusion into new regions, the striped field mouse has become an alien species there. *Apodemusagrarius* causes significant harm to agriculture and is one of the most important pests of grain crops. In tree nurseries, *A.agrarius* destroys seeds of valuable tree species and gnaws at the bark of saplings of broadleaf species and berry bushes. It is one of the most epidemiologically important rodents, involved in the circulation of the causative agents of haemorrhagic fever with renal syndrome (HFRS) and many other zoonotic infections. The foregoing allows us to classify the striped field mouse as a dangerous invasive alien species in the expanding part of the range. A lot of data accumulated for this species are of interest from both ecological and applied points of view. The accumulation and aggregation of data on the occurrence records of *A.agrarius* is relevant for the study of ecology, biogeography and construction of the spatial distribution and ecological niche models in the context of global climate change. We have created a dataset of 1603 occurrence records of this species, collected from 1936 to December 2020 by various zoologists, previously published or original. These records relate to a significant part of the striped field mouse’s range in Russia (1264 records) and neighbouring countries (339 records). The dataset shows the position of the northern and central parts of *A.agrarius* range, the disjunction of the range in Transbaikalia and isolated populations in the north of the range. The data were obtained in different formats from literature, indicating different degrees of accuracy of geographic coordinates and with several variations of the species' name. In the process of aggregating and fixing errors, we created a set of georeferenced occurrence records, adopted a controlled vocabulary, removed duplicates and standardised the format of records using unified data structure. We examined the dataset for inconsistencies with the taxonomic position of *A.agrarius* and removed the incorrect records. This paper presents the resulting dataset of *A.agrarius* occurrence records in the territory of Russia and neighbouring countries in a standardised format.

**New information:**

This is a validated and comprehensive dataset of occurrence records of *A.agrarius*, including both our own observations and records from literature. This dataset is available for extension by other researchers using a standard format in accordance with Darwin Core standards. In different countries, there are a lot of occurrence records for the striped field mouse, but the overwhelming part of them is presented in separate literary sources, stored in the form of maps and in zoological collections. Prior to this project, such information was not available to a wide range of researchers and did not allow the use of these spatial data for further processing by modern methods of analysis, based on geographic information systems (GIS technologies). The created dataset combines species occurrence records of many Soviet zoologists who studied the distribution of the striped field mouse over a significant part of its recent range, in Russia and neighbouring countries (within the former USSR). The final set of records was created by combining the species occurrence records using a uniform data structure, checking geographic coordinates and removing duplicate and erroneous records. The dataset expands the available information on the spatial and temporal distribution of the dangerous invasive species in Russia and neighbouring countries of the former USSR (Estonia, Latvia, Lithuania, Belarus, Ukraine, Moldova, Georgia, Azerbaijan, Kazakhstan and Kyrgyzstan).

## Introduction

**Figure 1.**
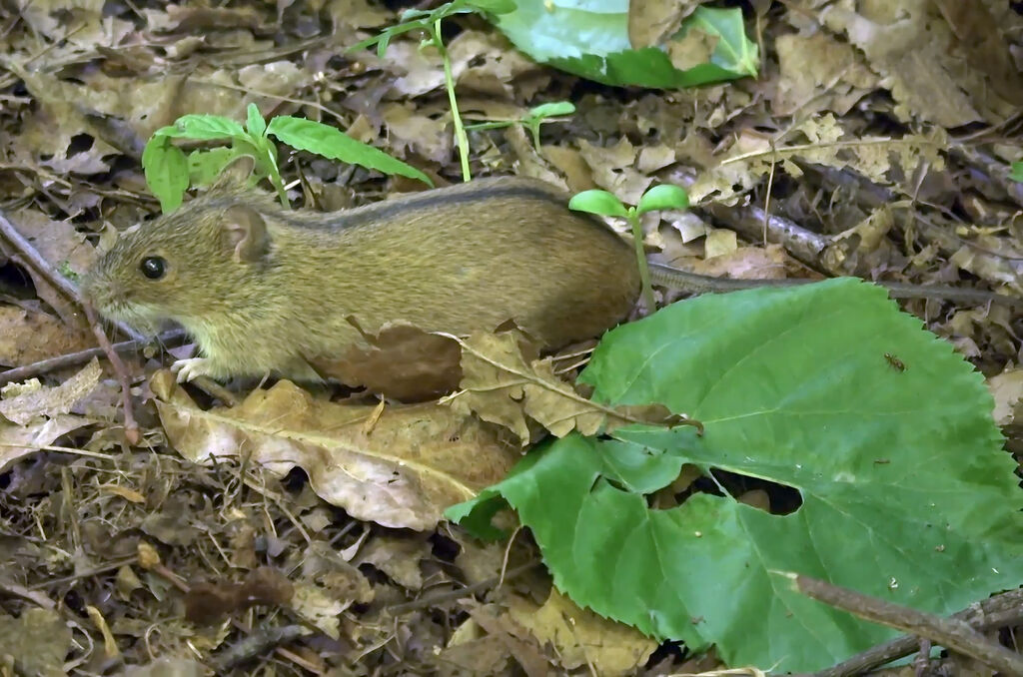
Striped field mouse (*Apodemusagrarius* Pallas, 1771) (Photo by Shmukler E., 29 May 2018, Losiny Ostrov National Park, Moscow).

*Apodemusagrarius* (Pallas, 1771) is the single representative of the subgenus Apodemus in the fauna of Russia (Wilson and Reeder 2005). Body length is up to 126 mm, tail length up to 90 mm (on average, about 70% of body length). By its general appearance, this species is easily distinguished from other Palearctic murids by a noticeable black dorsal stripe (Fig. 1). *Apodemusagrarius* is more tolerant of humid habitats than other mouse species within its range. Mixed feeding is typical. Seeds predominate, with their role increasing by autumn, the proportion of green food and insects is high and berries are less likely to be consumed (Okulova et al. 2011a, Okulova et al. 2011b). The species often reaches high densities. The maximum numbers were observed in the middle course of the Amur River and its tributary - Ussuri, the foothills of Altai and the Caucasus and the Volga Delta, where, during peak years, the abundance index was over 30 animals per 100 trap-days (Plater-Plohotsky 1936, Shkilev 1960, Kulik 1971, Tikhonova et al. 1992, Karaseva et al. 1999a, Okulova et al. 2011b, Okulova et al. 2011a). The seasonal breeding peak in the Central Black Soil Economic Region in Russia occurs in June-August and, in the Amur Region, in August. Average litter size in the Central Black Soil Economic Region varies from 4.7 to 7.8. Reproduction under the snow during winter was observed in the Tambov Region and the Amur Region (Okulova et al. 2012). The striped field mouse is characterised by high ecological plasticity, which allows it to adapt to human-modified environments thanks to a variety of mechanisms, including behavioural ones (Nikitina 1958, Khlyap et al. 1986, Moskvitina and Suchkova 1994, Agulova et al. 2008). It can rapidly spread along riparian corridors and over agricultural lands. It penetrates stored agricultural materials (hay, vegetables in containers etc.), with which it can be transported over long distances by humans; for example, it was accidentally introduced to the northern shores of the Sea of Okhotsk (Pereverzeva et al. 2017).

### Native range

Limits of the native range are not clear. It consists of two isolated parts: western (in Europe, Siberia and Kazakhstan) and eastern (in Russian Far East, China and Korea). Molecular genetic studies have shown that the eastern part of the range is considered to be more ancient (Suzuki et al. 2008) and that the westward expansion occurred relatively recently during the interglacial period in Late Pleistocene and later, the range became disjunct (Latinne et al. 2020). Hence, it follows that the formation of both parts of the range is not associated with human activity, i.e. does not apply to invasive processes in the modern understanding of this phenomenon. The native range in the western part apparently encompassed forest-steppe including foothills and mountainous areas. The forests, located to the north and the steppes located to the south, were not inhabited by the striped field mouse before their development by humans, because they are unsuitable for this species. A significant portion of the western native range is located in Russia. The Russian Far East segment of the eastern part of the range is located in the ancient part of the range (Suzuki et al. 2008, Latinne et al. 2020). It changed slightly in the 20^th^ century (see below) and, in our opinion, is almost entirely located within the native range of the striped field mouse. The contact zone between the native and recently-colonised range in China is unknown.

### Recent distribution

**Figure 2.**
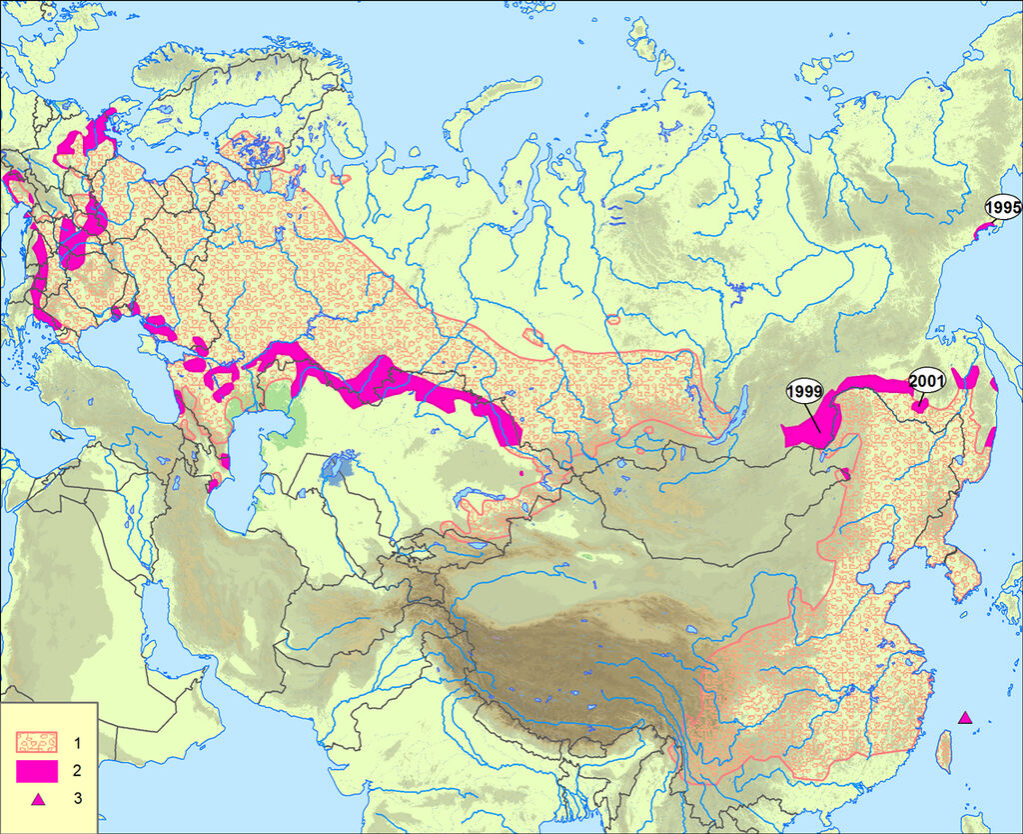
Dynamics of range of *Apodemusagrarius* (Dgebuadze et al. 2018) 1 – distribution in the first half in the 20^th^ century; 2 – range expansion over the second half of the 20^th^ and the beginning of 21^st^ centuries (according to Tikhonova et al. 1992 with additions); 3 – Senakaku (Uotsuri) Isles (Iwasa 2015). The numbers on the map indicate the years of striped field mouse invasions in the east of Russia.

The isolation of two large parts of the range is still recent. Up to the mid-20^th^ century, the western part extended from Central Europe to the upper Angara River (Kaneko et al. 2016, Dgebuadze et al. 2018). In the south, it reached the Caucasus and the Tien Shan foothills (Fig. 2). Northwards, it reached Finland, southern Karelia (up to the Kondopoga Town) and southern Arkhangelsk Region (Emelyanova et al. 2003); several isolated populations have been found near Arkhangelsk (Karaseva et al. 1992a). Westwards, it reached Germany and Italy (Mitchell-Jones et al. 1999, Dgebuadze et al. 2018). Records from France (GBIF.org 2018) require verification. Southwards, it spread to the northern Balkan Peninsula (Gliwicz and Kryštufek 1999, Spitzenberger and Engelberger 2014) and the European part of Turkey (Kefelioglu et al. 2003). There are records from Georgia and Azerbaijan (Karaseva et al. 1992a).

The eastern part of range previously extended from the Amur Region and southern Ussuriland, through Korea and much of eastern and southern China, reaching northern Myanmar (Kaneko et al. 2016). It occurs in Taiwan (possibly ancient distribution); a phenotypically distinctive and likely ancient population occurs on Senkaku Islands, north of Taiwan (Iwasa 2015). In recent decades, range expansion has been observed: west to Transbaikalia (Pavlenko et al. 2007, Bazhenov et al. 2015), north to the vicinity of Evoron Lake (Kartavtseva et al. 2011) and the northern shores of the Sea of Okhotsk (Dokuchaev et al. 2011, Pereverzeva et al. 2017). *Apodemusagrarius* have also been found in eastern Mongolia (Stubbe and Chotolchu 1968, Dulamtseren 1970).

### Pathways and vectors of invasions

The striped field mouse is an ancient agrophilic invader. With the beginning of human cultivation of land, mice began to populate cereal crops and associated weeds, where the number of mice became higher than in natural habitats (Tupikova et al. 2000, Neronov et al. 2001). As the forests were cut down for farm use, the striped field mouse moved north. It became a typical hemisynanthrope with increasing urbanisation (Kucheruk 1988, Karaseva et al. 1992a, Karaseva et al. 1992b, Khlyap and Warshavsky 2010, Tikhonova et al. 2012). The northern part of the modern range of the striped field mouse in Eastern Europe is the result of the first wave of invasion (ancient invasion) into arable lands, vegetable gardens and settlements. In our opinion, this part of the range was mainly formed from the beginning of the agricultural period to the 19^th^ century, as the forests were reduced and the northern regions were developed (Khlyap and Warshavsky 2010). However, mice could penetrate into certain regions even later. For example, it is believed that the striped field mouse invaded the previously underdeveloped region of the Volga and Western Dvina watershed in the late 1960s or the early 1970s (Istomin et al. 2013).

In the second half of the 20^th^ century, range expansion was noted in Germany, Italy, Austria, Czech Republic, Slovakia, Hungary, Slovenia, Ukraine (Gliwicz and Kryštufek 1999, Spitzenberger and Engelberger 2014, Kaneko et al. 2016, GBIF.org 2018), in Moldova, Azerbaijan and Kyrgyzstan (Dgebuadze et al. 2018). In Austria, the area colonised by the striped field mouse from 1996 to 2013 was 140 km long and 56 km wide (Spitzenberger 1997, Spitzenberger and Engelberger 2014). The greatest advance to the south, caused by the ploughing of virgin grasslands, was observed in the north of Kazakhstan. Range expansion of the striped field mouse in the Azov and Caspian Regions is also considered amongst invasions of the second half of the 20^th^ century (Tikhonova et al. 1992).

Invasion of the striped field mouse into new regions of eastern Russia was noted in Amur Region in the second half of the 20^th^ century (Tikhonova et al. 1992). It continued at the turn of the 20^th^ and 21^st^ centuries. In 1995, it was discovered on the northern shores of the Sea of Okhotsk (Dokuchaev et al. 2011). It was shown that it was accidentally introduced here from seaports of the southern Russian Far East and from China (Pereverzeva et al. 2017). Since 1999, mice can also be recorded in Transbaikalia (in 2001 – Pavlenko et al. 2007, Bazhenov et al. 2015); five individuals were captured in the vicinity of Evoron Lake (Khabarovsk Region) (Kartavtseva et al. 2011).

### Habitat

In undisturbed habitats, the striped field mouse usually inhabits floodplains with grassy vegetation and sparse forest, moist ravines and gullies, banks of water bodies covered with bushes, reeds, cattail and sedge (Dgebuadze et al. 2018). With the spread of agriculture, it colonises cereal fields, field margins and weed thickets, i.e. *A.agrarius* is a typical agrophile (Tupikova et al. 2000). Under the traditional harvesting system, in the autumn, it accumulated in ricks – long stacks of straw, hay or cereal crops. Such ricks, especially with unthreshed grain, were of significant importance for preserving the mouse population in the winter (Kulik 1951, Kucheruk and Rubina 1953). To the south in the steppe zone, the striped field mouse settles along the banks of irrigation canals and forest belts. Northwards distribution is associated with penetration into rural and urban settlements, where it prefers wastelands overgrown with weeds, parks, vegetable patches and gardens (hemisynanthrope). It is a typical inhabitant of public gardens and parks in many modern cities of European Russia and is able to inhabit the buildings themselves, mainly rural, without breaking ties with the surrounding habitats (Andrzejewski et al. 1978, Kucheruk 1988, Karaseva et al. 1999b, Karaseva et al. 1992b, Tikhonova et al. 2012). In the marginal parts of the range, such as the southwest of the Valdai Upland, the proportion amongst small mammals was 61% and 40% in the fields of cereals and in settlements, decreasing to 3% in upland and lowland meadows and to 0.1–0.3% in spruce forests and cutover lands (Istomin et al. 2013).

### Impact on other species, ecosystems and humans

The striped field mouse is one of the most important pests of crops and causes significant damage to agriculture, especially during peak years. In forest nurseries, it destroys the seeds of valuable tree species and nibbles the bark of saplings of broadleaf species and berry bushes. It pollutes and destroys agricultural produce in warehouses (Plater-Plohotsky 1936, Sviridenko 1949, Karlik 2008). The striped field mouse is the most important rodent reservoir of many zoonotic diseases, such as haemorrhagic fever with renal syndrome, often fatal to humans (Lee 2003). Of the hantaviruses, the Hantaan virus circulates in the striped field mouse populations in the Far East and the Dobrava/Belgrade, genotypes Kurkino and Saaremaa - in Eastern and Central Europe (Tkachenko et al. 2012, Klempa et al. 2012). In these Regions, the presence of striped field mice in settlements is very epidemiologically dangerous. In natural foci of leptospirosis, it is the most important carrier of *Leptospirakirschneri* (serovar Mozdok), the most intense foci of which, in Russia, are known in the North Caucasus (Karaseva and Kokovin 1965) and which is also found in Germany (Fischer et al. 2018). Striped field mouse is an important host of tick nymphs (Nikitina et al. 1960) and, therefore, participates in the circulation of tick-borne infections, such as tick-borne encephalitis. Striped field mouse is also involved in the circulation of tularaemia, lymphocytic choriominengitis, listeriosis, erysipeloid and other zoonotic diseases (Karaseva 1979, Shekhanov 1979) and the list of such diseases continues to grow (Kraljik et al. 2016, Gajda et al. 2017).

The primary data on occurrence of *A.agrarius* are important for ecological research and management, including the assessment of invasion risks, formulation of preventative and management plans in the context of global climate change and land use. These data are also important for the prediction of potential habitats of the species using modern methods of modleling ecological niches. Refined, validated and reformatted spatio-temporal distribution data can help prevent further spread of this invasive species.

Our goal was to combine the accumulated knowledge and expertise of historical data to create a validated, publicly available dataset in a modern format on occurrence records in Russia and neighbouring countries. To achieve this goal, the following tasks were undertaken: 1) To preserve and ensure the availability of the results of the fieldwork of many Soviet zoologists who have studied the distribution of striped field mouse in Russia and other countries of the former USSR; 2) To map the distribution records of the striped field mouse over a significant part of its range; 3) To provide data on the expansion of the species’ range in Russia and neighbouring countries for the second half of the 20^th^ century and the beginning of the 21^st^ century and 4) To create a valid dataset for modelling the ecological niche of the species and its dynamics for the entire range or its large parts.

## General description

### Purpose

The striped field mouse is included in the TOP-100 list of the most dangerous invasive species in Russia (Dgebuadze et al. 2018), which also includes organisms from various groups: bacteria, chromists, fungi, vascular plants, alveolates, ctenophores, nematodes, molluscs, arthropods (crustaceans and insects) and chordates (ascidians; ray-finned fishes, amphibians, reptiles, birds and mammals). In recent decades, due to global climate and land use changes, the species’ range has expanded, which makes the analysis of distribution data an urgent and valuable task. We aggregated and curated occurrence records to study and confirm the distribution of this species in Russia and neighbouring countries (Khlyap et al. 2021). These records are also important for the development of ecological niche models to study the correlation between climate and land use variables and the presence of striped field mice through space and time. The publication of occurrence records will provide valid information and will contribute to the continuation of research of the invasion process, based on aggregated data in a standardised format.

## Project description

### Title

Aggregated occurrence records of the invasive alien striped field mouse (*Apodemusagrarius* Pall.) in Russia and neighbouring countries.

### Study area description

The study area covers most of the distribution range of the striped field mouse (*A.agrarius*). In Russia, the westernmost records lie in Kaliningrad Region, the northernmost in Arkhangelsk Region, the easternmost in Magadan Region and the southernmost in Dagestan Republic. The study area also includes the territories of neighbouring countries: Estonia, Latvia, Lithuania, Belarus, Ukraine, Moldova, Georgia, Azerbaijan, Kazakhstan and Kyrgyzstan. The dataset includes occurrence records on the northern and north-eastern limits of the species range, the southern limit of distribution in the Caucasus and characterises the central part of the species range. These records also show a disjunction between the western and eastern parts of the range, which lies between Lake Baikal and the Upper Amur Basin.

## Sampling methods

### Sampling description

Striped field mouse occurrence records were collected from various sources: field data gathered by Soviet zoologists over 40 years, including ~ 20,000 capture records; collections in zoological museums (the Zoological Museum of Moscow State University, the Museum of the Zoological Institute of the Russian Academy of Sciences in St. Petersburg and the Siberian Zoological Museum in Novosibirsk); records obtained by Epidemiology Control Stations of Russia; literature data (Tembotov 1972, Karaseva et al. 1992a, Karaseva et al. 1992b, Khe 2002, Emelyanova et al. 2003, Okulova et al. 2005, Oparin and Oparina 2006, Pavlenko et al. 2007, Kartavtseva et al. 2011, Bolshakov et al. 2015, Pereverzeva et al. 2017); and original fieldwork data.

A significant part of the occurrence records (1453) was obtained from the map "Distribution of striped field mouse in the USSR", published by Karaseva et al. (Karaseva et al. 1992a, Karaseva et al. 1992b). In that map, each data point has its individual number with information on the location, time of collecting, habitat, abundance of the species and data source. The dataset was expanded by adding 118 records from the Caucasus and Ciscaucasia (Tembotov 1972, Okulova et al. 2005); a record from Arkhangelsk Region in northern Russia, where the striped field mouse has appeared no later than in the late 19^th^ century (Emelyanova et al. 2003); three records from the Russian westernmost part of the range (Oparin and Oparina 2006, Bolshakov et al. 2015) and 18 records from the eastern part of the range (Pavlenko et al. 2007, Kartavtseva et al. 2011, Pereverzeva et al. 2017). In addition, we included our own captures of striped field mice (10 original records) in the Regions not covered by the above publications. Thus, the integrated dataset contains 1603 records from the territory of the former USSR (Table 1).

Literature data covering the time interval 1936-2020 (Table 1) were analysed and digitised. Three types of occurrence records were included in the dataset. Records of the first type include geographic coordinates in literature or in our own data. Records of the second type have only location maps without exact coordinates. For this data type, geographic coordinates of occurrence records were approximated after linking the maps to the base vector maps of Russia and the USSR, with a choice of at least 20 reference points in the ArcGIS Desktop 10.4. The base maps for the USSR and Russia were obtained from the open source Open Street Map (https://www.openstreetmap.org). Records of the third type contained sufficient descriptions of the collection sites to determine the exact geographic coordinates using Google Earth. If geographic coordinates were not originally specified in decimal degrees, they were converted to decimal degrees using the WGS84 datum.

We used the data presented in Tikhonova et al. (Tikhonova et al. 1992) to include in our dataset information on the dynamics of the striped field mouse range in the second half of the 20^th^ century. All occurrence records from Karaseva et al. (Karaseva et al. 1992b), falling into the range expansion over the second half of the 20^th^ century (Fig. 2), we dated 1951\1992.

### Quality control

When analysing and checking specimens, only those for which the location could be determined were included in the dataset. A significant part of the records were taken from publications authored or verified by leading Russian zoologists (VN Bolshakov, LV Frisman, EV Karaseva, IV Kartavtseva, VV Kucheruk, IL Kulik, NA Nikitina, NM Okulova, MV Pavlenko, AK Tembotov and GN Tikhonova) to ensure correct identification of the specimens. The striped field mouse is easy to distinguish from other rodents of the Palearctic fauna by its colour pattern and morphology. It is a black dorsal stripe, clearly visible against the greyish-ochre colour of the back fur (Fig. 1). Geographic coordinates of occurrence records that were incorrectly georeferenced were changed to represent coordinates consistent with the locality listed in the occurrence metadata. We analysed a number of important columns in accordance with the Darwin Core specification when creating the dataset:

baseOfRecord: Data records with an unknown baseOfRecord were removed from our dataset to ensure that all accepted records were based on observation data.

ScientificName: The striped field mouse was originally described by Pallas (1771) as *Musagrarius* Pallas, 1771 and there are occurrence records in literature by different names due to the existence of synonyms and invalid subspecific designations. Wilson and Reeder (Wilson and Reeder 2005) listed 25 subspecies of *A.agrarius*. Seven of them occur in the former USSR: *mantchuricus* Thomas, 1898; *karelicus* Ehrström, 1914; *ognevi* Johansen, 1923; *septentrionalis* Ognev, 1924; *tianschanicus* Ognev, 1940; *caucasicus* Kuznetzov, 1944; *volgensis* Kuznetzov, 1944 (Gromov et al. 1963, Pavlinov and Khlyap 2012). In our dataset, various taxonomic names of striped field mouse are given using the oldest valid name - *Apodemusagrarius* Pallas, 1771.

eventDate: Our dataset concerns an invasive species and it was important to reflect the timing of invasions. According to the Darwin Core specification, the column indicates the time interval during which the event — the appearance of the striped field mouse — took place. Analysis of literature only made it possible to divide that information on the first records into three time intervals (cases). The first case corresponds to the interval 1936/1992, which means that the species was first recorded between 1936 and 1992 and remains present now (there is no data on extinction). The second case corresponds to the interval 1951/1992, which means that the species appeared between 1951 and 1992. For the third case, the specific year after 1992 is used. The separation of the date records into time intervals is important for characterizing the regions of invasion of the striped field mouse.

Data within columns were edited using controlled vocabulary and Darwin Core standards. Primary data were retained when controlled vocabulary could not be utilised. Spelling or transcription errors were noted and changed to reflect the correct spelling of the species. We removed duplicate records from multiple sources by removing occurrences with identical eventDates. Corrected data were formatted according to Darwin Core standards (Wieczorek et al. 2012).

## Geographic coverage

### Description

**Figure 3.**
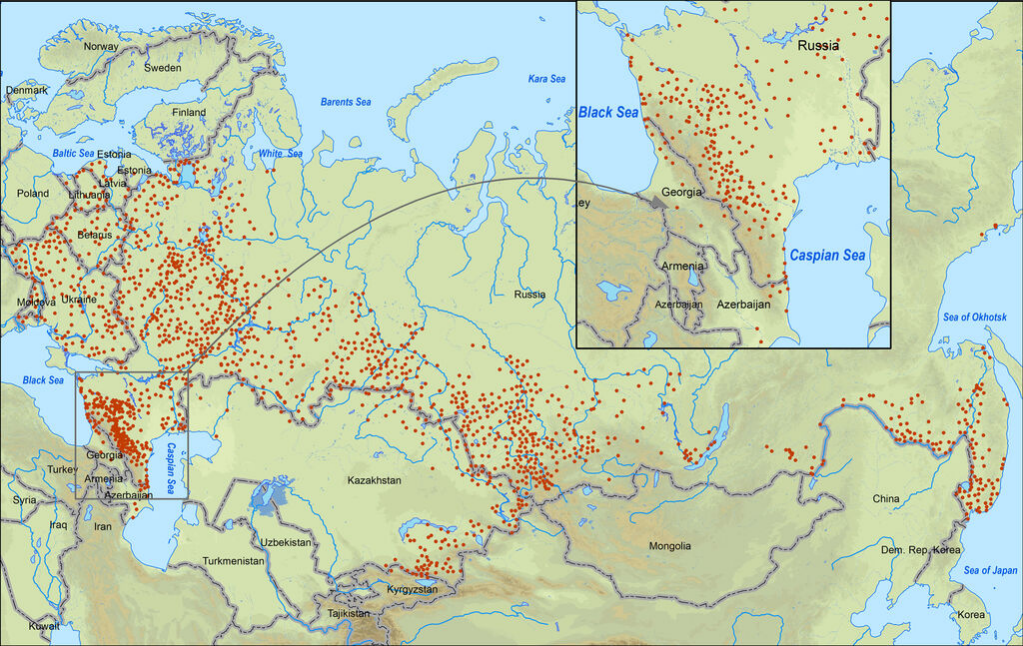
Occurrence records of *Apodemusagrarius* in the former USSR.

The geographic range of the dataset covers the territory of Russia and neighbouring countries within the borders of the former USSR (Estonia, Latvia, Lithuania, Belarus, Ukraine, Moldova, Georgia, Azerbaijan, Kazakhstan and Kyrgyzstan) (Fig. 3). The most northern (Arkhangelsk Region, 64.8069 N, 40.5975 E), western (Kaliningrad Region, 54.6003 N, 21.2661 E) and eastern (Magadan Region, 59.7368 N, 150.9325 E) occurrence records are from Russia. The southernmost occurrence records located in Russia are from Dagestan Republic (41.6238 N, 47.8926 E) and the southernmost record in the entire dataset is from Azerbaijan (39.1271 N, 49.1041 E).

A significant part of the occurrence records 78.9% (1264) are located in Russia, in the Baltic countries bordering on Russia: Estonia, Latvia, Lithuania 1.5% (24) records are registered, in the eastern European countries of former USSR - Belarus, Ukraine and Moldova, there are 10% (161) records, in the south-western part of the range on the territory of Georgia and Azerbaijan 1.2% (20) records are registered, in the territory of Asian countries - Kazakhstan and Kyrgyzstan, there are a relatively large number of records 8.3% (134) (Table 2).

### Coordinates

39.1271 and 64.8069 Latitude; 21.2661 and 150.9325 Longitude.

## Taxonomic coverage

### Description

This dataset is devoted to one species of Rodentia in the family Muridae (*Apodemusagrarius*).

## Temporal coverage

### Notes

**Figure 4.**
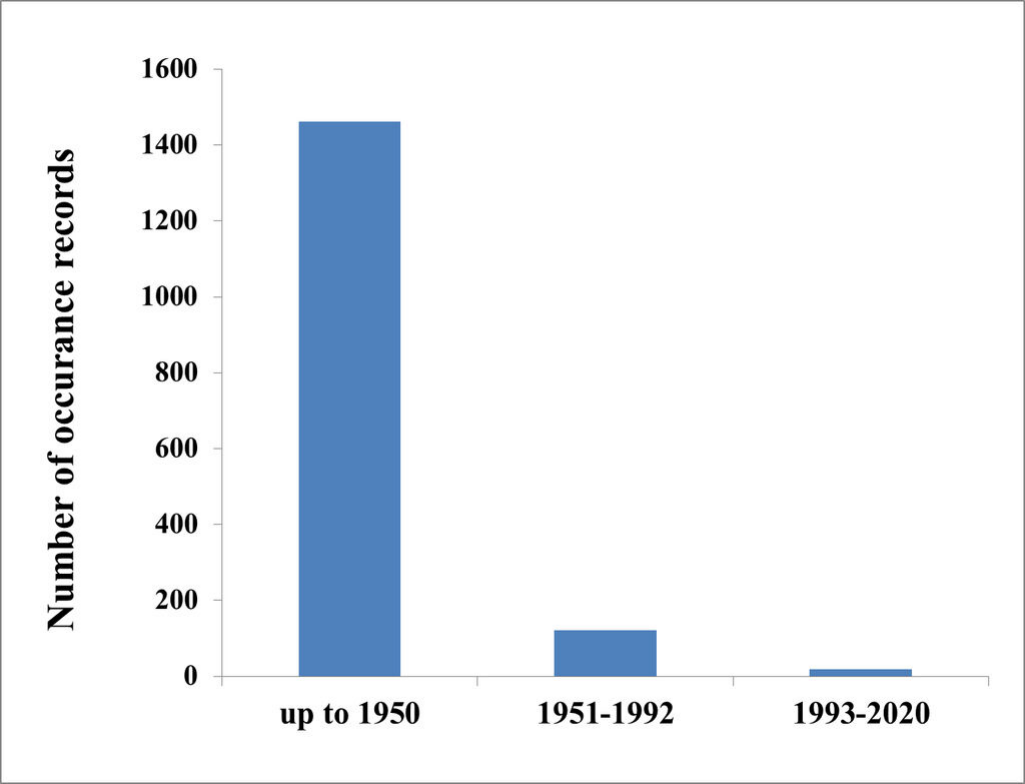
Temporal profile of the number of *Apodemusagrarius* occurrence records that were documented.

01-01-1936 through to 31-12-2020 (Fig. 4).

## Usage licence

### Usage licence

Other

### IP rights notes

**IP rights notes**:

See individual records for usage rights.

## Data resources

### Data package title

Aggregated occurrence records of the invasive alien striped field mouse (*Apodemusagrarius*) in the former USSR.

### Number of data sets

1

### Data set 1.

#### Data set name

Striped field mouse (*Apodemusagrarius*) occurrences in Russia and adjacent countries according to published data.

#### Data format

Darwin Core Archive

#### Number of columns

22

#### Download URL

https://www.gbif.org/dataset/4071501e-25fd-4a1c-9381-a3d5cb8d166c

#### Data format version

1.2

#### Description

Data are formatted according to Darwin Core standards (http://rs.tdwg.org/dwc/terms) and the column labels and column descriptions are based on this standard.

**Data set 1.** 

Column label	Column description
id	The name or acronym in use by the institution having custody of the object(s) or information referred to in the record.
bibliographicCitation	A bibliographic reference for the resource as a statement indicating how this record should be cited (attributed) when used. Any data records that were edited cite this data paper in this column.
basisOfRecord	The specific nature of the data record. We used a Darwin Core controlled vocabulary for our basisOfRecord that included "HumanObservation".
occurrenceID	In this dataset, occurrence records use the ID number from its holding facility when applicable. Occurrence records that did not have a unique ID were given their own unique observation ID.
occurrenceRemarks	Comments or notes about the occurrence.
eventDate	The date-time or interval during which an Event occurred. For occurrences, this is the data-time when the event was recorded.
habitat	A category or description of the habitat in which the Event occurred.
fieldNumber	An identifier given to the event in the field. Often serves as a link between field notes and the Event.
eventRemarks	Comments or notes about the Event.
countryCode	The standard code for the country in which the Location occurs.
stateProvince	The name of the next smaller administrative region than country (state, province, canton, department, region etc.) in which the Location occurs.
county	The full, unabbreviated name of the next smaller administrative region than stateProvince (county, shire, department etc.) in which the Location occurs.
locality	The specific description of the place. Less specific geographic information can be provided in other geographic terms (higherGeography, continent, country, stateProvince, county, municipality, waterBody, island, islandGroup). This term may contain information modified from the original to correct perceived errors or to standardise the description.
verbatimLocality	The original textual description of the place.
decimalLatitude	The latitude of the location from which the catalogued item was collected, expressed in decimal degrees.
decimalLongitude	The longitude of the location from which the catalogued item was collected, expressed in decimal degrees.
geodeticDatum	The ellipsoid, geodetic datum or spatial reference system (SRS) upon which the geographic coordinates given in decimalLatitude and decimalLongitude are based. Recommended best practice is use of the EPSG code as a controlled vocabulary to provide an SRS, if unknown. Otherwise, use of a controlled vocabulary for the name or code of the geodetic datum, if unknown.
coordinateUncertaintyInMetres	The horizontal distance (in metres) from the given decimalLatitude and decimalLongitude describing the smallest circle containing the whole of the Location. Leave the value empty if the uncertainty is unknown, cannot be estimated or is not applicable (because there are no coordinates). Zero is not a valid value for this term.
georeferenceSources	A list (concatenated and separated) of maps, gazetteers or other resources used to georeference the Location, described specifically enough to allow anyone in the future to use the same resources.
scientificName	The full scientific name.
kingdom	The full scientific name of the kingdom in which the taxon is classified.
taxonRank	The taxonomic rank of the most specific name in the scientificName.

## Figures and Tables

**Table 1.** Occurrence records sources of striped field mouse. The total number of occurrences (1603) obtained from each source are listed as of December 2020.

Source	Number of records
Tembotov 1972	61
Karaseva et al. 1992b	1453
Khe 2002	52
Emelyanova et al. 2003	1
Okulova et al. 2005	5
Oparin and Oparina 2006	1
Pavlenko et al. 2007	13
Kartavtseva et al. 2011	1
Bolshakov et al. 2015	2
Pereverzeva et al. 2017	4
Original records (2017-2020)	10

**Table 2.** The number of *Apodemusagrarius* occurrence records across the study area as of December 2020.

Country	Nuber of provinces /regions	Number of records
Estonia	1	1
Latvia	7	14
Lithuania	9	9
Belarus	6	36
Ukraine	24	116
Moldova	9	9
Georgia	6	11
Azerbaijan	7	9
Kazakhstan	10	129
Kyrgyzstan	2	5
Russia	72	1264
